# Cardiac output‐mediated regulation of cerebral blood flow during exercise: Clinical perspectives on the indirect impact of muscle metaboreflex

**DOI:** 10.1113/EP091591

**Published:** 2024-03-18

**Authors:** Shigehiko Ogoh

**Affiliations:** ^1^ Department of Biomedical engineering Toyo University Kawagoe Japan

**Keywords:** cardiac output, exercise, exercise pressor reflex, metaboreflex

## Abstract

The muscle metaboreflex stimulates the elevation of arterial blood pressure, aiming to rectify the oxygen deficit by enhancing oxygen delivery to support muscle activity. Moreover, activating the muscle metaboreflex significantly increases cardiac output (CO) by increasing factors such as heart rate, ventricular contractility, preload, stroke volume and mobilization of central blood volume. Previous studies indicate that ageing and cardiovascular diseases modify the muscle metaboreflex during exercise, limiting the ability to increase CO during physical activity. Alongside reduced exercise capacity, the attenuated rise in CO due to abnormal muscle metaboreflex in these patients impedes the increase in cerebral blood flow during exercise. Considering that CO plays a pivotal role in regulating cerebral blood flow adequately during exercise, this occurrence might contribute to an elevated risk of cerebral diseases, and it could also, at least, reduce the effective role of exercise in preventing cerebral disease and dementia among elderly individuals and patients with cardiovascular conditions. Therefore, it is important to consider this phenomenon when optimizing the effectiveness of exercise rehabilitation in patients with cardiovascular disease to prevent cerebral diseases and dementia.

## Abstract (highlights)

- **What is the topic of this review?**
  This review focuses on the role of muscle metaboreflex‐induced augmentation of cardiac output during exercise in patients with cardiovascular disease.
- **What advances does it highlight?**
  Sufficient regulation of cardiac output is crucial for maintaining cerebral perfusion. Cerebral hypoperfusion might play a pivotal role in the development of brain disease in patients with heart failure. This review highlights the impact of the metaboreflex on the exercise‐induced rise in cardiac output and explores its related pathophysiology.

## 1 INTRODUCTION

During exercise, the critical redistribution of blood to active muscles relies on significant cardiovascular and autonomic adjustments. These adaptations involve increased cardiac output (CO), determined by heart rate (HR) and stroke volume (SV), elevated arterial blood pressure (ABP), and reduced vascular resistance caused by metabolism in the active muscles. Coordinating the regulation of these haemodynamic factors involves both muscle metabolism and the nervous system (central command, exercise pressor reflex and baroreflex), integrating information from various sources (Fadel, 2015). Regarding muscle blood flow, it is crucial to note that partial metabolism in active muscles takes precedence over the influence of sympathetic nerve activity (SNA), leading to vasodilatation. This phenomenon, known as ‘functional sympatholysis’, contrasts with the vasoconstriction caused by an increase in SNA in inactive muscles. Therefore, these two physiological factors play a role in determining muscle blood flow, ultimately influencing SV through both preload and afterload mechanisms. The motor cortex (central command) and muscle receptors (exercise pressor reflex) primarily stimulate the elevation of ABP and CO. Meanwhile, receptors in the aortic, carotid, heart and pulmonary arteries (arterial and cardiopulmonary baroreflexes) collectively oversee these adjustments. Although an increased HR directly contributes to heightened CO through central command activation, delineating its effects on other CO factors is challenging due to the intricate nature of this interaction.

During exercise, an important physiological mechanism entails a reflexive increase in blood pressure triggered by the accumulation of metabolites due to insufficient oxygen delivery (relative hypoperfusion) stimulating skeletal muscle afferents. This reflex, known as the muscle metaboreflex, aims to rectify the oxygen deficit for muscle contraction by enhancing oxygen delivery to sustain muscle activity (Augustyniak et al., 2001; Hansen et al., 1994; O'Leary & Sheriff, 1995; O'Leary et al., 1999; Spranger et al., 2013). Conversely, previous studies have shown that the activation of the muscle metaboreflex leads to significant increases in CO by affecting factors such as the rise in HR, ventricular contractility, preload, SV and mobilization of central blood volume (Amann et al., 2011; Bastos et al., 2000; Crisafulli et al., 2011, 2003; Ichinose et al., 2010; O'Leary, 1993; O'Leary & Augustyniak, 1998; O'Leary et al., 2019; Sala‐Mercado et al., 2006; Spranger et al., 2013). This review focuses on the impact of metaboreflex on the exercise‐induced rise in CO that affects cerebral blood flow (CBF) regulation and delves into its associated pathophysiology.

### 1.1 EXERCISE‐INDUCED REGULATION OF CO VIA MUSCLE METABOREFLEX

During exercise, increasing CO is essential to enhance blood flow to active muscles, vital for physical activity. This heightened CO is achieved through escalated HR and enhanced myocardial contractility, under the influence of both autonomic modulation from the central command and the exercise pressor reflex. The central command sets a sympathetic activity baseline and reduces vagal tone, correlating with exertion intensity and motor signals from the cortex (McCloskey & Mitchell, 1972; O'Leary, 1993; Strange et al., 1993; Thornton et al., 2002). While it is acknowledged that central command escalates HR and blood pressure by increasing sympathetic activity and reducing parasympathetic tone, its activation mainly leads to heightened CO during exercise via increased HR. Yet, there is insufficient evidence showing its direct impact on cardiac contractility, preload or afterload – factors influencing CO during exercise. The challenge lies in distinguishing haemodynamic changes attributed solely to central command amidst the influence of the exercise pressor reflex. Further research is crucial for a comprehensive understanding.

The exercise pressor reflex finely modulates initial autonomic activation by responding to signals from mechanoreceptors and metaboreceptors, nerve endings within muscles. It regulates sympathetic activity based on mechanical and metabolic conditions (Crisafulli et al., 2003; Iellamo et al., 1999; Nobrega et al., 2014; O'Leary, 1993; Piepoli et al., 1995). Specifically, muscle contractions during exercise trigger the muscle metaboreflex through type III and IV sensory afferents stimulated by metabolic by‐products, inducing a reflexive increase in blood pressure (Alam & Smirk, 1937; Coote et al., 1971). This elevation in blood pressure can arise from increased CO, heightened vascular resistance in inactive muscles (affected by exercise‐induced increase in muscle SNA), or a combination of both (Fisher et al., 2015; Grotle et al., 2020; Kaur et al., 2015; Mark et al., 1985; Spranger et al., 2013). Autonomic regulation of CO involves the modulation of HR through inputs from both sympathetic and vagal nerves at the sinoatrial node, while sympathetic innervation across the ventricles significantly influences cardiac contractility (Coote & Chauhan, 2016). However, HR often returns to baseline after post‐exercise muscle metaboreflex activation despite the increased sympathetic activity, indicating that the heightened CO during muscle metaboreflex activation is likely attributable to increased SV (Barrett‐O'Keefe et al., 2018; Boyes et al., 2023).

**FIGURE 1:**
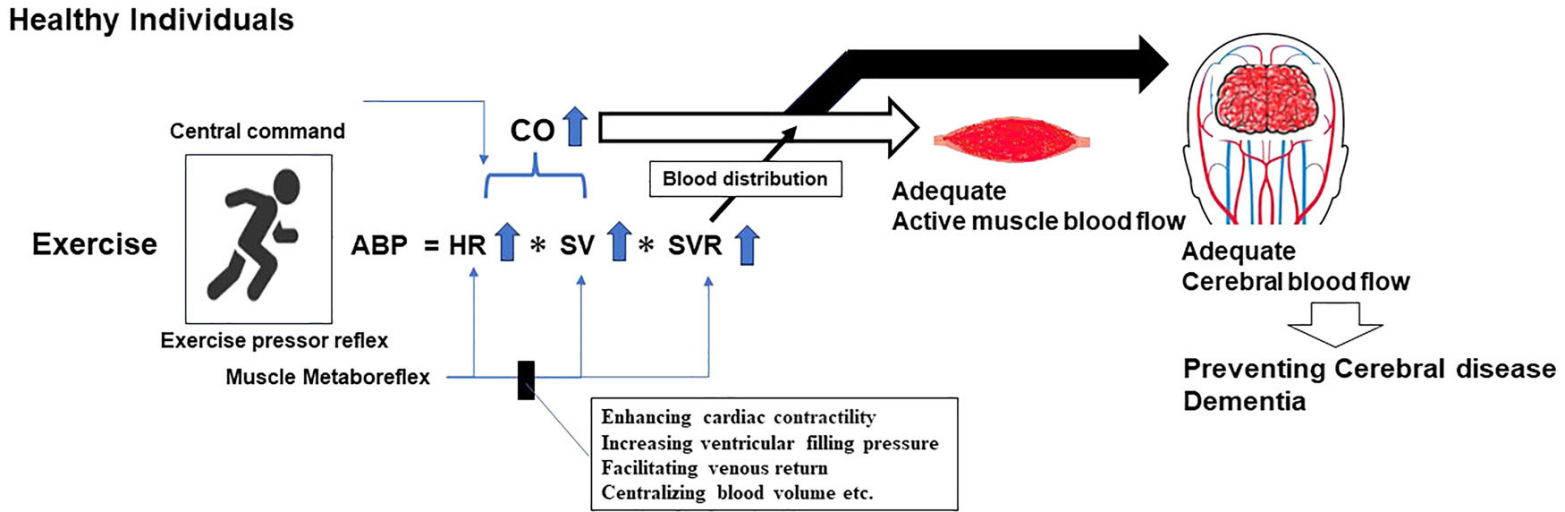
The role of muscle metaboreflex in regulating blood flow in active muscles and the brain in healthy individuals.

Ventricular performance significantly influences the cardiac response during dynamic exercise and the ability to sustain exercise workload. An essential reflex that facilitates heightened CO during exercise is the muscle metaboreflex (Crisafulli et al., 2003, 2011; Hammond et al., 2000; Kaur et al., 2015; O'Leary & Augustyniak, 1998; O'Leary et al., 1999, 2019; Sheriff et al., 1998) (Figure 1). Several previous studies have shown the impact of the muscle metaboreflex on cardiac contractility, influencing preload, SV and ventricular performance (Amann et al., 2011; Bastos et al., 2000; Crisafulli et al., 2007, 2003, 2009, 2011; Ichinose et al., 2010; Marongiu et al., 2013; Milia et al., 2015; Nobrega et al., 2014; O'Leary & Augustyniak, 1998; Sala‐Mercado et al., 2006; Spranger et al., 2013). Moreover, the metaboreflex triggers vasoconstriction, thereby increasing ventricular filling pressure, facilitating venous return and centralizing blood volume. This action supports increases in both SV and CO during exercise (Bastos et al., 2000; Crisafulli et al., 2009; O'Leary et al., 2019; Sheriff et al., 1998). These alterations consequently enhance CO, improving perfusion to the actively engaged skeletal muscles (Crisafulli et al., 2003, 2011; Hammond et al., 2000; Kaur et al., 2015; O'Leary & Augustyniak, 1998; O'Leary et al., 1999, 2019; Sala‐Mercado et al., 2006). Additionally, baroreflexes contribute to regulating metaboreflex‐induced enhanced sympathetic activity during exercise, ensuring consistent blood pressure by adjusting muscle vasodilatation and HR, and maintaining a balance between vascular resistance and cardiac function (Fadel et al., 2001; Raven et al., 2006; Sheriff, 2006). These findings indicate exercise‐induced change in physiological factors such as central command, the exercise pressor reflex and baroreflex significantly impact the determination of sufficient CO during exercise through autonomic function. Specifically, the haemodynamic response to metaboreflex activation is a complex and integrated phenomenon involving HR, cardiac performance, preload and afterload. Yet, this mechanism is important to achieve the typical cardiovascular response to exercise, particularly in healthy individuals.

### 1.2 MUSCLE METABOREFLEX AND ABNORMAL CO REGULATION DURING EXERCISE IN PATHOPHYSIOLOGY

Changes in muscle metaboreflex activation were observed in patients with heart failure and spinal cord injuries and in individuals with diastolic dysfunction (Crisafulli et al., 2009, 2007; Milia et al., 2015; Piepoli et al., 2008). Therefore, abnormal cardiovascular responses during exercise in these patients may be due to changes in the muscle metaboreflex because it is associated with preload and cardiac contractility, influencing cardiovascular function (Amann et al., 2011; Bastos et al., 2000; Crisafulli et al., 2007, 2003, 2009, 2011; Ichinose et al., 2010; Marongiu et al., 2013; Milia et al., 2015; Nobrega et al., 2014; O'Leary & Augustyniak, 1998; Sala‐Mercado et al., 2006; Spranger et al., 2013). For example, patients with heart failure demonstrate a restricted ability to augment CO during exercise due to decreased SV via metaboreflex‐induced reflexive tachycardia (Augustyniak et al., 2006; Crisafulli et al., 2007; Hammond et al., 2000; Kaur et al., 2018; O'Leary et al., 2004). Metaboreflex‐induced vasoconstriction elevates ventricular filling pressure, supporting venous return and leading to a form of blood volume ‘centralization’ essential for supporting SV and CO (Bastos et al., 2000; Sheriff et al., 1998). In fact, a decrease in ventricular filling rate has been noted to impede the metaboreflex‐induced SV response in patients with spinal cord injury. In addition, the heart failure condition attenuates the sensitivity of baroreflex control of HR, despite causing baroreflex resetting to a higher HR. This indicates the possibility that this attenuation of baroreflex function may lead to abnormal cardiovascular responses during exercise in these patients (Iellamo et al., 2007). In healthy individuals, the baroreflex function curve is reset to higher operating points without a change in sensitivity, whereas exercise‐induced resetting of baroreflex function is caused by the muscle metaboreflex (Fadel et al., 2003; Ogoh et al., 2003, 2002; Ogoh, Brothers et al., 2005; Ogoh, Fisher et al., 2005; Raven et al., 2006). Indeed, the heart failure condition enhances the muscle metaboreflex, and this enhanced muscle metaboreflex attenuates the sensitivity of baroreflex control of HR (Iellamo et al., 2007). These findings suggest that abnormal cardiovascular responses during exercise in these patients may be attributed to a reduction in CO control during exercise via attenuated baroreflex function.

Crisafulli et al. (2009) demonstrated that patients with spinal cord injuries displayed a reduced blood pressure elevation in response to the metaboreflex compared to healthy individuals. In these patients, the ability to increase SV, ventricular filling rate and venous return in response to the metaboreflex was limited, resulting in a constraint in augmenting CO during exercise. Moreover, Milia et al. (2015) found that activation of the metaboreflex led to significantly lower preload in elderly individuals compared to younger individuals, despite a higher blood pressure response to the metaboreflex. Additionally, it has been reported that the exercise‐induced increase in CO is attenuated in the elderly compared to middle age (O'Connor et al., 2015). The influence of muscle metaboreflex activation on the ventricular relaxation rate was notably reduced in heart failure (Mannozzi et al., 2021). Thus, the reduced capacity to increase CO during muscle metaboreflex activation in heart failure may not solely stem from diminished ventricular contraction but also from alterations in ventricular relaxation and diastolic function. Since exercise capacity is attenuated in patients with heart failure (Borlaug, 2014; Omote et al., 2022), these findings clearly suggest that the ability of the heart to regulate CO adequately via metaboreflex, rather than relying solely on cardiovascular reserve, is essential for efficiently performing even submaximal exercise. On the other hand, in patients with type 2 diabetes mellitus (DM2), the CO response to exercise decreased due to ageing and diabetes (O'Connor et al., 2015; Roberto et al., 2019). Moreover, individuals with DM2 exhibited a notable increase in systemic vascular resistance (SVR), indicating intensified arteriolar vasoconstriction during metaboreflex activation compared to healthy controls. The inability to augment SV and CO was attributed to diminished venous return, impaired cardiac function and heightened afterload in patients with DM2 (Roberto et al., 2019). Within this cohort, it is evident that the blood pressure response to the metaboreflex primarily depends on the amplification of SVR rather than adaptations in cardiac performance.

These previous studies have shown that ageing and cardiovascular diseases modify the muscle metaboreflex during exercise, restricting the ability to increase CO during physical activity. Such limitations in enhancing CO during exercise could potentially curtail overall exercise capacity. It is widely acknowledged that exercise training or chronic exercise for the elderly and patients with cardiovascular diseases increases baseline CBF and cognitive function and, consequently, helps prevent dementia and other brain diseases (Kleinloog et al., 2019). Therefore, within our current discussion, in these populations where the muscle metaboreflex is attenuated, it is crucial to note that the impact of exercise rehabilitation on cardiovascular regulation may differ from that observed in healthy individuals in terms of preventing cardiovascular disease.

### 1.3 CO PLAYS A CRUCIAL ROLE IN ESTABLISHING CBF AS WELL AS EXERCISE CAPACITY DURING EXERCISE

While the influence of SNA on regulating CBF during exercise remains a topic of controversy (Ogoh, 2008; Ogoh & Ainslie, 2009a, 2009b; Brassard et al., 2017), some previous studies, akin to cardiovascular adjustments, have suggested that the sympathetic activation induced by the muscle metaboreflex might contribute to regulating CBF during exercise (Braz et al., 2014; Friedman et al., 1991, 1992; Jorgensen et al., 1993; Ogoh et al., 2019; Prodel et al., 2016; Vianna et al., 2009). For example, it has been reported (Friedman et al., 1991, 1992; Jorgensen et al., 1993) that blocking afferent muscle fibres with local anaesthesia eliminated the exercise‐induced rise in CBF, suggesting the muscle metaboreflex affects cerebral blood vessels. Another study (Braz et al., 2014) showed the muscle metaboreflex's impact on CBF was masked by isometric exercise‐induced hypocapnia via hyperventilation using a carbon dioxide (CO_2_) clamp to create low CO_2_ conditions. Also, Ogoh et al. (2019) demonstrated that activation of metabolically sensitive skeletal muscle afferent fibres alters the regulation of CBF in both the posterior and the anterior cerebral circulation during isometric exercise. On the contrary, Jorgensen et al. (1992) showed that cerebral perfusion during exercise mirrors enhanced brain activation, which appears to be unrelated to the activation of muscle metaboreceptors. Hence, further investigations may be necessary to delineate the precise impact of direct autonomic regulation triggered by the muscle metaboreflex on the regulation of CBF during exercise, especially considering the inconsistent findings from prior studies. More interestingly, it has been reported that cardiac vagal nerve activity increases during exercise and is associated with the regulation of coronary artery blood flow, as indicate in animal models (Shanks et al., 2023). This finding suggests that exercise‐induced vagal activity may contribute to the increase in CBF during exercise, but this is just speculation.

Importantly, CO is another physiological factor directly linked to establishing CBF (Ogoh & Ainslie, 2009a, 2009b). Ogoh, Brothers et al. (2005) demonstrated a linear association between CO and CBF in healthy human volunteers, both at rest and during exercise. This relationship persisted independently of arterial partial pressure of carbon dioxide ($P_{\mathrm{aC}O_{2}}$) and cerebral autoregulation. However, the relationship between changes in CBF and CO induced by central blood volume was attenuated during exercise compared to rest. Moreover, Ogoh et al. (2007) demonstrated that as exercise intensity rises, the decline in cardiac–arterial baroreflex function at its operating point does not significantly impact the dynamic control of CBF, even in scenarios where the exercise‐induced rise in CO is diminished through cardiac β1‐adrenergic blockade. This observation suggests an increased involvement of arterial baroreflex‐mediated regulation of CO in maintaining CBF during exercise, emphasizing its role in reflexively governing the systemic vasculature. In any case, these findings suggest that CO plays an important role in establishing CBF during exercise as well as rest (Ogoh & Ainslie, 2009a, 2009b; Ogoh, Fisher et al., 2005).

Furthermore, it is essential to consider the impact of exercise‐induced cerebral metabolism on establishing CBF. It is widely acknowledged that cerebral metabolism significantly influences regional CBF through neural coupling. Consequently, regional CBF, as measured by functional magnetic resonance imaging, serves as an index of partial cerebral neural activity. An earlier study by Linkis et al. (1995) investigated the mean blood velocity in both the right and left anterior and middle cerebral arteries during exercises involving the right and left foot and hand. The study demonstrated a notable increase in blood flow velocity in the artery supplying the cortical projection of the exercising limb. However, it is important to note that the cerebral metabolic ratio during exercise remains unaffected by variations in CBF (Dalsgaard et al., 2004). This phenomenon is attributed to the increased extraction of oxygen and glucose from the blood in the cerebral circulation, which determines cerebral neural activity as the primary fuel source. Despite the elevated cerebral neural activity during intense dynamic exercise, CBF decreased to baseline levels, as observed in a study by Sato et al. (2011). It is worth mentioning that the evaluation of neural coupling during a visual task involves changes in posterior cerebral artery blood velocity (Phillips et al., 2016) but is not attributed to exercise‐induced neural coupling. Under these backgrounds, while the increase in regional CBF induced by cerebral metabolism contributes to the overall CBF in larger arteries, its impact is limited due to the small vascular bed of the regional cerebral area. Additionally, cerebral metabolism may not be a strong factor determined by CBF, even though CBF is a crucial factor for cerebral neural activity (Ogoh & Ainslie, 2009a; Ogoh et al., 2022).

**FIGURE 2:**
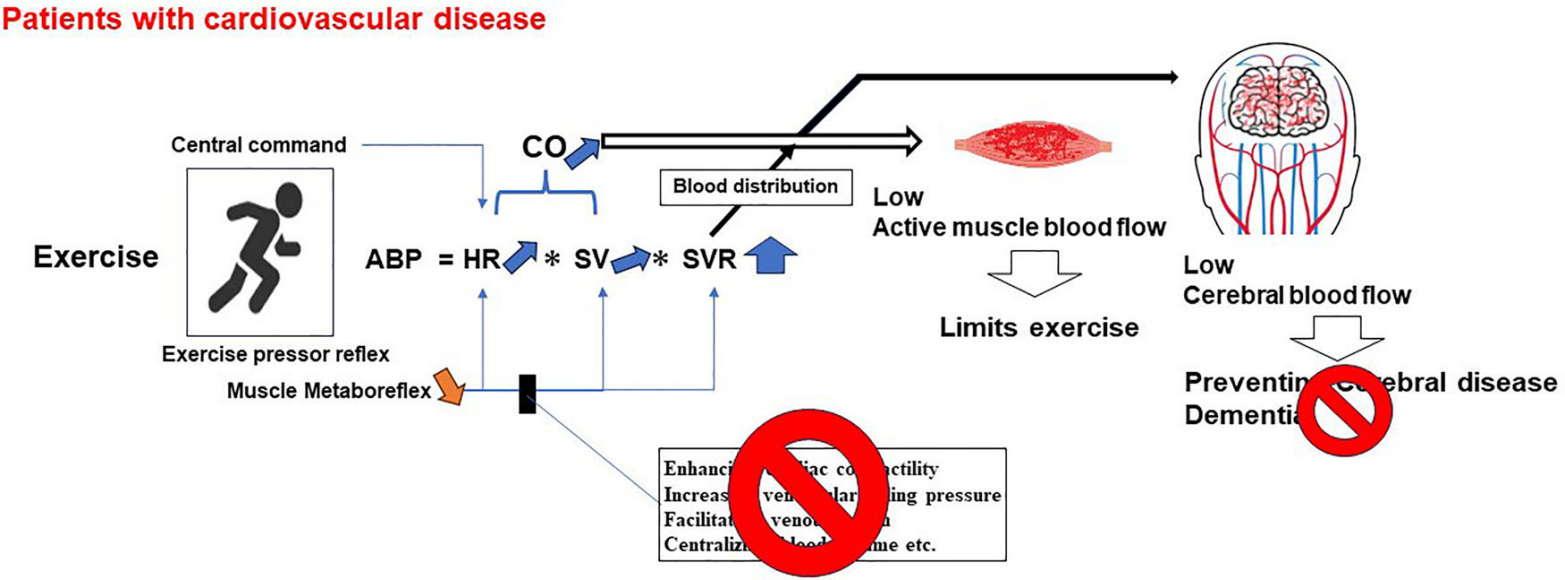
In patients with cardiovascular disease, attenuated activation of muscle metaboreflex results in limited exercise performance due to inadequate muscle blood flow. Additionally, the exercise‐induced increase in cerebral blood flow is diminished owing to a reduction in cardiac output. Consequently, exercise rehabilitation aimed at preventing cerebral disease and dementia may induce reduced cerebral vascular stimulation in patients with cardiovascular disease, potentially resulting in differing effects compared to those observed in healthy individuals.

Under this background, alongside reduced exercise capacity, the subdued rise in CO through abnormal muscle metaboreflex in patients with cardiovascular disease might limit the increase in CBF during exercise (Figure 2). This phenomenon might be associated with the reduced effectiveness of exercise rehabilitation in preventing cerebral diseases and dementia for patients with cardiovascular disease. This is attributed to inadequate cerebral vascular stimulation due to reduced CBF associated with abnormal CO responses. Moreover, this occurrence might contribute to an increased risk of cerebral diseases among elderly individuals and patients with cardiovascular diseases (Haeusler et al., 2011; Hassell et al., 2013; Kim & Kim, 2015; Writing Group et al., 2010), as they experience reduced increases in CO during exercise.

### 1.4 DOES ABNORMAL REGULATION OF CO INDUCED BY CARDIOVASCULAR DISEASE POSE A RISK OF CEREBRAL DISEASE?

Systemic diseases, such as heart failure – a leading cause of morbidity and mortality – can significantly impact other organ systems, notably the brain (Kim & Kim, 2015). Several studies suggest that heart failure increases the risk of cognitive dysfunction and stroke (Haeusler et al., 2011; Hassell et al., 2013; Writing Group et al., 2010). However, the mechanism underlying heart failure (HF)‐induced brain disease remains unclear. Haeusler et al. (2011) have described the possible pathophysiological factors contributing to brain impact from cardiac dysfunction including HF‐induced left ventricular hypokinesia (Pullicino et al., 2000), hypoperfusion (Roman, 2004) or hypotension (Pullicino et al., 2009) as direct factors, and impairments in cerebrovascular reactivity (Georgiadis et al., 2000), endothelial dysfunction (Lip & Gibbs, 1999) and related elements as indirect factors.

Importantly, in severe chronic HF patients, global CBF is reduced by 14−30% (Rajagopalan et al., 1984) and HF patients with low CBF were nearly 2.5 times more likely to die or require urgent transplantation during a median follow‐up period of 3 years (Kim & Kim, 2015). These findings suggested that cerebral hypoperfusion may be key to causing brain disease in HF patients. Significantly, the reduction in exercise‐induced CO through β1‐blockade has been found to mitigate the rise in CBF during exercise, as demonstrated by Ide et al. (2000). Furthermore, documented cases in patients with heart failure (Hellstrom et al., 1997) and atrial fibrillation (Ide et al., 1999) also support the notion that a diminished exercise‐induced increase in CO restricts the corresponding increase in CBF during exercise. On the other hand, Choi et al. (2006) reported a decreased global CBF at rest in patients with advanced heart failure (according to the New York Heart Association (NYHA) classification). However, they demonstrated that this reduction in CBF was not reliant on cardiac dysfunction in these heart failure patients, including factors such as exercise capacity (heart volume) or left ventricular ejection fraction (cardiac function). Therefore, the diminished CBF in heart failure patients might result from indirect physiological factors induced by heart failure rather than direct cardiac dysfunction. The lack of correlation between cardiac dysfunction and abnormal CBF response could be associated with compensatory mechanisms aimed at maintaining adequate CBF against abnormal perfusion due to cardiac dysfunction (cardiac output) or perfusion pressure (blood pressure). Therefore, the reduced establishment of adequate CO during exercise induced by the attenuated metaboreflex might indirectly diminish CBF regulation during exercise. This limitation might potentially be linked to an elevated risk of cerebral disease and dementia in patients with cardiovascular disease, impacting the effectiveness of exercise rehabilitation in preventing such conditions. Further investigations are imperative to elucidate the mechanisms underlying cardiovascular disease‐induced cerebral diseases, including dementia.

## 2 CONCLUSIONS

Activating the muscle metaboreflex adequately increases CO for performing exercise, and consequently affects CBF. This suggests that the activation of the muscle metaboreflex during exercise influences CBF indirectly. However, ageing and cardiovascular diseases modify the muscle metaboreflex during exercise, limiting the ability to increase CO and consequently reducing exercise capacity. Additionally, this diminished capacity to increase CO restricts the attainment of sufficient CBF during exercise. Therefore, understanding the role of the metaboreflex is crucial for exercise rehabilitation aimed at preventing dementia and cerebral diseases, especially in the elderly and in patients with cardiovascular diseases.

## AUTHOR CONTRIBUTIONS

Sole author.

## CONFLICT OF INTEREST

The author declares no conflict of interest.
